# The relationship between Internet use and loneliness of middle-aged and older adult people: the moderating effect of residence

**DOI:** 10.3389/fpubh.2024.1284180

**Published:** 2024-01-31

**Authors:** Qiusha Li, Chunxiao Yang, Zixuan Zhao, Chenxiao Yang, Zhongming Chen, Dongmei Huang, Wenqiang Yin

**Affiliations:** ^1^School of Public Health, Shandong Second Medical University, Weifang, Shandong, China; ^2^School of Management, Shandong Second Medical University, Weifang, Shandong, China

**Keywords:** Internet use, loneliness, middle-aged, older adult, digital divide

## Abstract

**Objectives:**

The proportion of middle-aged and older adult people exposed to the Internet continues to grow. Internet use may have an impact on the mental health of the older adult, especially loneliness. This study analyzed the relationship between Internet use and presence of loneliness.

**Methods:**

A total of 550 person aged 45 years and above were randomly selected from a province in eastern China at the end of 2022. The outcome variable was presence of loneliness, as measured by self-report. Descriptive analysis, chi-square test and binary logistic analysis were used to analyze the data.

**Results:**

58.3% of respondents use the Internet. Internet use could reduce the possible of reported loneliness in middle-aged and older adult people (*OR* = 0.652, 95%*CI:* 0.465, 0.940), and residence played a moderating role in the relationship between them. Middle-aged and older adults who used the Internet for 1–3 h (*OR* = 0.464, 95%*CI*: 0.275, 0.784) and 3–5 h (*OR* = 0.484, 95%*CI*: 0.247, 0.946) were less likely to felt lonely than those who used the Internet for less than 1 h per day. In addition, middle-aged and older adult people using the Internet to contact relatives and friends (*OR* = 0.488, 95%*CI*:0.292, 0.818), read the news (*OR* = 0.485, 95%*CI*:0.277, 0.848), assets management (*OR* = 0.297, 95%*CI*:0.109, 0.818) were less likely to report loneliness, while those who made online payment (*OR* = 3.101, 95%*CI*:1.413, 6.807) were more likely to report loneliness.

**Conclusion:**

There is a significant negative correlation between Internet use and presence of loneliness, but different Internet duration and content have different effects on loneliness in middle-aged and older adult people. We should pay attention to the impact of Internet use on loneliness in middle-aged and older adult people.

## Introduction

As China’s economy grows and medical services improve, people’s life expectancy increases, and China is entering an aging society at the fastest rate (1–3). China has become the country with the largest older adult population in the world, accounting for about a quarter of the world’s older adult population (4). The World Health Organization regards 45 years old and 60 years old as the age criteria for middle-aged and older adult people, respectively (5). According to China’s seventh census, there will be more than 600 million people aged 45 and above in China in 2020, accounting for about 42.6% of the total population (6). With the rapid development of Internet technology, the proportion of the middle-aged and older adult population in the Internet community continues to grow. Studies have shown that the proportion of people aged 45 to 59 who have access to the Internet in China has reached 50.0% (7). This part of the “quasi-older adult” will be an important part of the future older adult population in China. The proportion of Internet users aged 60 and above has also climbed from 6.7% in March 2020 to 11.3% in 2022. Especially during the COVID-19 pandemic, when the Internet has brought many conveniences to life and Internet usage has risen sharply (3, 8, 9). At the same time, the mental health of middle-aged and older adult people may be affected by Internet use (10). Loneliness is an important indicator of the mental health of the older adult, which refers to the negative experience when there is a difference between the expected social relationship and the established social relationship (11). Studies have found that the older adult are vulnerable to loneliness due to the loss of social roles, the reduction of economic resources and the increase of injury (12). Loneliness may lead to an increased risk of depression, cognitive impairment, reduced life satisfaction, and severe loneliness is even associated with a higher risk of death (13–17).

The impact of the Internet on middle-aged and older adult people, especially on loneliness, has attracted more and more attention. However, the academic community has not yet reached a unified conclusion on the relationship between Internet use and loneliness of middle-aged and older adult people. Some scholars believe that by using the Internet, the older adult can break the restrictions of geographical space and enhance their connection with the outside world. Especially for the middle and young older adult, the use of the Internet can improve social participation and have a positive impact on mental health (18–20). Shillair found that the use of the Internet can maintain and enrich the social relations of the older adult, reduce the impact of loneliness, and improve life satisfaction (21). Lam, Wang and others found that the use of the Internet can reduce the anxiety, depression and other negative emotions of the older adult (22, 23). Cotten also found that older people who are reluctant to socialize also benefit from Internet use (24). Some scholars hold different views that Internet use will occupy the social time of the older adult in real life. And unhealthy Internet behavior reduces the opportunities for face-to-face communication, which is not conducive to emotional expression and the maintenance of social relations, thus increasing inner loneliness (25–28). Other scholars have also pointed out that people are affected differently by the content and duration of Internet use (29). In terms of online time, most of the existing studies focus on teenagers or college students (30, 31), and there are few studies on the relationship between online time and loneliness in middle-aged and older adult groups. Although some studies have explored the association between Internet use and loneliness and reported different results (18, 27, 28), there is limited evidence on the relationship between Internet use duration and content and loneliness. In this paper, examining the roles of Internet use duration and content may help understand the conflicting findings in the literature.

In addition, the digital divide between urban and rural areas in China is huge, and there may be obvious differences between urban and rural residents (2). At present, although the Internet is promoted nationwide, there are more non-Internet users in rural areas (32). A study on China shows that with the acceleration of China’s urbanization process, the rural youth population continues to flow to the city, and the number of rural empty nesters is increasing. Compared with cities, the mental health problems of the older adult in rural areas are more prominent (33). Previous studies have shown that during the pandemic, when middle-aged and older adult people have certain restrictions on their activities, they can actively connect with others through the use of the Internet to reduce loneliness. However, the differences in Internet use between urban and rural areas may have different effects on the loneliness of middle-aged and older adult people in urban and rural areas (34). In this study, we will explore the following questions: What is the relationship between Internet use and presence of loneliness among middle-aged and older adults during the COVID-19 pandemic? Specifically, what are the effects of Internet use duration and content on loneliness among middle-aged and older adult people? Considering the existence of urban–rural differences, is the relationship between Internet use and loneliness of middle-aged and older adult people influenced by place of residence?

## Methods

### Study design and sampling

This study is a cross-sectional survey conducted in Shandong Province in December 2022. The respondents were middle-aged and older adult people aged 45 and above. Shandong Province is an economically developed region in the east of China. It is the second most populous province in China. In this study, we used the method of stratified random sampling to collect data. In order to ensure that the survey samples are representative of Shandong Province. Firstly, we selected three prefecture-level cities in Shandong Province according to the level of economic development, which are economically developed cities, economically medium cities and economically underdeveloped cities. Secondly, according to the same principle, three counties (cities, districts) were extracted from each prefecture-level city, and three natural villages (communities) were extracted from each county. Finally, we randomly selected 20 middle-aged and older adult people aged 45 and above in each natural village (community). To protect the privacy of the survey subjects, we follow the principle of informed consent and voluntary participation. The questionnaires were filled in anonymously by individuals on site. We assigned four to six trained investigators to each site, who answered questions in real-time during the investigation. If participants were unable to fill out the questionnaire independently, the investigators were available to offer face-to-face assistance. At the end of the investigation, the questionnaires were collected on the spot, investigators were checked by themselves and checked each other. Invalid questionnaires such as too many missed answers, logical errors and skipping answers errors were eliminated. In the end, a total of 550 questionnaires were sent out, and 518 valid questionnaires were recovered, with an effective recovery rate of 94.18%. Written informed consent was obtained from all participants in this study. This study was approved by the Ethics Committee of Weifang Medical University.

### Measurement

The dependent variable of the study was presence of loneliness. We used one question to measure the loneliness of middle-aged and older adult people during the epidemic. Respondents were asked to answer “Do you feel lonely?” The answer is “yes” and “no,” where 1 is “yes” and 0 is “no.” We simply defined loneliness as: loneliness is a feeling and experience of subjectively being isolated and estranged from others or society. At the scene of the investigation, the investigator gives a detailed explanation. The independent variables were Internet use (“Do you use the Internet?”; 1 = yes, 0 = no), length of Internet use (“How long do you use the Internet every day?”; 1 = less than 1 h, 2 = 1–3 h, 3 = 3–5 h, 4 = more than 5 h), and content of Internet use. Content of Internet use included contacting relatives and friends, watching the news, watching videos, getting health codes (a QR code used to provide personal health information during the epidemic prevention and control period), searching for health information, online payment, online shopping, playing games, and managing money (27, 32, 35). In addition, we collected the following characteristics of the respondents as control variables: gender (1 = male, 2 = female), age (1 = under 60 years old, 2 = 60 years old and above), marital status (1 = not married, 2 = married), residence (1 = rural, 2 = urban), education level (1 = primary school and below, 2 = junior high school, 3 = senior high school and above), employment status (1 = inactive, 2 = active).

### Statistical analysis

SPSS software was used to process the data. Firstly, the basic demographic characteristics and loneliness of the respondents were analyzed descriptively. Secondly, the chi-square test was used to compare the differences of loneliness among people with different characteristics. Thirdly, taking loneliness as the dependent variable, and taking Internet use, Internet use length and Internet use content as the independent variables, the relationship between Internet use and loneliness of middle-aged and older adult people was tested by binary logistic regression model, and the significance level was set as *p* < 0.05. Finally, we used Model 1 of the PROCESS program in SPSS to analyze the moderating effect. In Model 1, presence of loneliness was taken as the dependent variable, Internet use as the independent variable, residence as the moderating variable, and other variables as the control variable, to test the moderating effect of residence on the relationship between Internet use and presence of loneliness.

## Results

### Socio-economic and demographic characteristics of respondents and presence of loneliness

A total of 518 middle-aged and older adult people were investigated, including 227 males, accounting for 43.8%, and 291 females, accounting for 56.2%. There were 286 people under 60 years old, accounting for a 55.2%, and 232 people over 60 years old, accounting for a 44.8%. There were 450 persons (86.9%), with the status of being married. 408 people (79.2%) live in rural areas. There are 197 people with primary school education and below, accounting for 38.0%, and 191 people with high school education and above, accounting for 36.9%. The employment status was active in respect of 331 persons (63.9%).

Of the 518 middle-aged and older adult people, 240 felt lonely, accounting for 46.3%. The results of the chi-square test are shown in Table 1. The presence of loneliness of the interviewees varied with marital status (*p* < 0.05) and educational level (*p* < 0.05). The proportion of participants who were not married felt lonely was higher than those who were married, and the proportion of participants with primary school education or below felt lonely was higher than those with junior high school education, senior high school education or above.

**Table 1:** Sociodemographic characteristics and the presence of loneliness among total study group.

Variables	Total *N*	Lonely *N* (%)	Not lonely *N* (%)	*χ^2^*	*p*
**Gender**					
Male	227	106 (46.7%)	121 (53.3%)	0.022	0.929
Female	291	134 (46.0%)	157 (54.0%)		
**Age**					
<60	286	127 (44.4%)	159 (55.6%)	0.953	0.332
≥60	232	113 (48.7%)	119 (51.3%)		
**Marital status**					
Not married	68	42 (61.8%)	26 (38.2%)	7.497	0.009
Married	450	198 (44.0%)	252 (56.0%)		
**Residence**					
Rural	408	183 (44.9%)	225 (55.1%)	1.909	0.191
City	107	56 (52.3%)	51 (47.7%)		
**Education level**					
Primary school and below	197	98 (49.7%)	99 (50.3%)	6.190	0.045
Junior middle school	130	48 (36.9%)	82 (63.1%)		
High school and above	191	94 (49.2%)	97 (50.8%)		
**Employment status**					
Unemployed	187	81 (43.3%)	106 (56.7%)	1.071	0.314
Employed	331	159 (48.0%)	172 (52.0%)		
**Internet use**					
Yes	302	127 (42.1%)	175 (57.9%)	5.333	0.025
No	216	113 (52.3%)	103 (47.7%)		
**Internet duration**					
less than 1 h	122	68 (55.7%)	54 (44.3%)	9.664	0.022
1–3 h	146	55 (37.7%)	91 (62.3%)		
3–5 h	62	25 (40.3%)	37 (59.7%)		
More than 5 h	28	14 (50.0%)	14 (50.0%)		
**Internet content**					
Connect with friends and family	212	84 (39.6%)	128 (60.4%)	23.205	0.006
Watch the news	175	65 (37.1%)	110 (62.9%)		
Watch the video	174	65 (37.4%)	109 (62.6%)		
Get a health code	172	63 (36.6%)	109 (63.4%)		
Search for health information	132	53 (40.2%)	79 (59.8%)		
Online payment	93	46 (49.5%)	47 (50.5%)		
Online shopping	87	37 (42.5%)	50 (57.5%)		
Play games	34	15 (44.1%)	19 (55.9%)		
Assets management	33	11 (33.3%)	22 (66.6%)		

### Internet usage status of respondents

Among the respondents, 302 person use the Internet, accounting for 58.3%, and 216 person do not use the Internet, accounting for 41.7%. Among the middle-aged and older adult people who use the Internet, the majority of them spend 1–3 h online every day, with 146 people, accounting for 40.8%. Most people use the Internet to communicate with relatives and friends, with 212 people, accounting for 19.6%. In addition, our study found that middle-aged and older groups differed in Internet use, duration, and content. The results of the chi-square test showed that the presence of loneliness of respondents varied with Internet use (*p* < 0.05), Internet content (*p* < 0.05), and Internet duration (*p* < 0.05). The proportion of participants who were not use internet felt lonely was higher than those who were used, and the participants who spent less than 1 h online were more likely to felt lonely than those who spent more than 5 h, 3–5 h, and 1–3 h. The proportion of participants who used the Internet for online payments felt lonely was higher than those who used it for other purposes (see Table 1).

### The relationship between Internet use and presence of loneliness

We used a logistic regression model to examine the relationship between Internet use and presence of loneliness. Loneliness was the dependent variable (0 = no, 1 = yes), and Internet use was the independent variable, which were included in the logistic regression model. Model 1 in Table 2 reports results without control variables. Model 2 reports results with control variables. As shown in Model 1, middle-aged and older adult people who use the Internet were less likely to felt lonely (*OR* = 0.661, 95%*CI*:0.465, 0.940). This conclusion is still supported when control variables are included. As shown in Model 2, we found that marital status and employment status may be related to the presence of loneliness in middle-aged and older adult people. Married respondents were less likely to report loneliness (*OR* = 0.455, 95%*CI*:0.263, 0.787), and employed middle-aged and older adult people were more likely to report loneliness (*OR* = 1.585, 95%*CI*:1.034, 2.430). However, when we stratified the middle-aged and older adult samples, we found that the effect of Internet use on loneliness in middle-aged and older adult people was not significant, which we believe may be due to insufficient sample size.

**Table 2:** Effects of Internet use on loneliness.

Variables	Dependent variable: loneliness (0 = no, 1 = yes)		
	Model 1	Model 2	Model 3
	*OR* (95%*CI*)	*OR* (95%*CI*)	*OR* (95%*CI*)
Internet use (ref = No)	0.661 (0.465, 0.940)**	0.652 (0.465, 0.940)**	0.618 (0.418, 0.913)**
Gender (ref = Male)		0.975 (0.675, 1.408)	0.967 (0.418, 0.913)**
Age (ref = <60)		1.276 (0.818, 1.990)	1.338 (0.857, 2.092)
Marital status (ref = Not married)		0.455 (0.263, 0.787)***	0.453 (0.262, 0.785)***
Education level (ref = Primary school and below)		0.998 (0.774, 1.286)	0.981 (0.760, 1.266)
Employment status (ref = Unemployed)		1.585 (1.034, 2.430)**	1.575 (0.760, 1.266)**
Residence (ref = Rural)		1.539 (0.944, 2.510)	1.934 (1.112, 3.363)**
Internet use × Residence			0.295 (0.098, 0.888)**
Constant	1.097	0.713	1.117
*R*^2^	0.014	0.050	0.063

**p < 0.05, ***p < 0.01.

### The influence of Internet use duration and content on presence of loneliness

We used a logistic regression model to further examine the relationship between the duration and content of Internet use and loneliness. The presence of loneliness was taken as the dependent variable (0 = no, 1 = yes), the duration of Internet use (model 4) and the content of Internet use (model 5) were taken as the independent variables to be included in the logistic regression model. As shown in Table 3, compared with middle-aged and older adult people who used the Internet for less than 1 h per day, those who used the Internet for 1–3 h (*OR* = 0.464, 95% *CI*: 0.275, 0.784) and 3–5 h (*OR* = 0.484, 95%*CI*: 0.247, 0.946) were less likely to felt lonely. There was no statistically significant effect when the duration of use was more than 5 h. In addition, different contents of Internet use have different effects on middle-aged and older adult people. Using the Internet to contact relatives and friends (*OR* = 0.488, 95%*CI*:0.292, 0.818), read the news (*OR* = 0.485, 95%*CI*:0.277, 0.848), assets management (*OR* = 0.297, 95%*CI*:0.109, 0.818) middle-aged and older adult people were less likely to report loneliness, while those who made online payment (*OR* = 3.101, 95%*CI*:1.413, 6.807) were more likely to report loneliness.

**Table 3:** Effects of duration and content of Internet use on loneliness.

变量	Dependent variable: loneliness (0 = no, 1 = yes)					
	Model 4			Model 5		
	*OR*	95%*CI*	*P*	*OR*	95%*CI*	*p*
**Internet usage time (ref = less than 1 h)**						
1–3 h	0.464	0.275, 0.784	0.004			
3–5 h	0.484	0.247, 0.946	0.034			
More than 5 h	0.623	0.261, 1.490	0.288			
**Internet usage content**						
Connect with friends and family				0.488	0.292, 0.818	0.006
Search for health information				1.022	0.567, 1.842	0.942
Watch the news				0.485	0.277, 0.848	0.011
Watch the video				0.615	0.368, 1.026	0.063
Play games				1.976	0.726, 5.380	0.183
Assets management				0.297	0.109, 0.812	0.018
Get a health code				1.117	0.598, 2.087	0.728
Online payment				3.101	1.413, 6.807	0.005
Online shopping				0.699		0.361
Control variable	YES			YES		
Constant	1.662		0.583	1.076		0.938
R^2^	0.112			0.187		

### Moderating role of place of residence between Internet use and loneliness

**Figure 1:**
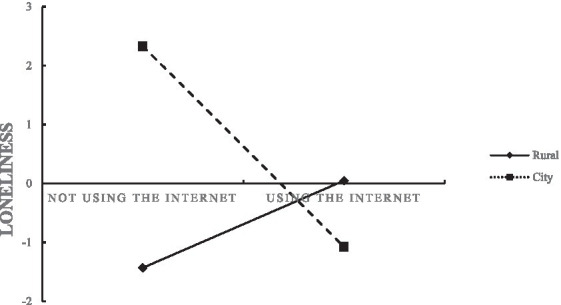
Discussion of the moderating role of residence.

We used the presence of loneliness as the dependent variable, Internet use as the independent variable, residence as the moderating variable, and carried out the moderating effect analysis on the basis of controlling other variables. Model 3 in Table 2 showed that the interaction terms of Internet use and residence significantly predicted loneliness (*p* < 0.05, *OR* = 0.295, 95%*CI*: 0.098, 0.888). A further simple slope test, as shown in Figure 1, showed that Internet use was not a significant predictor of loneliness in middle-aged and older adult people living in rural areas (*p* > 0.05, *OR* = 0.815, 95%*CI*: 0.536, 1.237), and in urban areas. Internet use was a significant predictor of loneliness among middle-aged and older adult people (*p* < 0.05, *OR* = 0.229, 95% *CI*: 0.080, 0.655). Internet use had a more significant effect on loneliness among middle-aged and older adult people in urban areas relative to those in rural areas.

## Discussion

This study investigated the current situation of Internet use in the middle-aged and older adult people and the impact of whether to use the Internet, the content of use and the duration of use on presence of loneliness. We learned about the Internet use behavior of middle-aged and older adults during the COVID-19 pandemic. The results of the study can further elucidate the relationship between Internet use and presence of loneliness in the middle-aged and older adult people, and provide reference for the development of interventions to improve the well-being of them.

First, in terms of Internet use, 58.3% respondents use the Internet. It is higher than the Internet access rate of the older adult aged 60 and over in China (38.6%) (1). However, compared with young people, the proportion is still low (36). Although the Internet usage rate of middle-aged and older adult people is relatively low, the proportion of middle-aged and older adult Internet users is increasing year by year. With regard to the content of Internet use, consistent with the research of Loipha et al., the needs of the older adult for the Internet are relatively simple, and most of them use it to contact relatives and friends, watch news, watch videos, search for health information, pay online and obtain health codes during the epidemic (37, 38). Studies have shown that older people are interested in the Internet primarily for health reasons and are more likely to search for health information online than other age groups (39, 40). In addition, the number of Internet applications in China has increased steadily, with the most obvious growth in short video, online payment and online shopping, which has also affected the daily life of middle-aged and older adult people to a certain extent (41). With regard to the duration of Internet use, the results of our study showed that most of the older adult use the Internet for less than 3 h a day, which is not much different from the survey results of the 51st Statistical Report on the Development of Internet in China (41), and is lower than that of the older adult in the United States (42).

Second, there is a significant negative correlation between Internet use and loneliness in middle-aged and older adult people, and residence plays a moderating role in the relationship between them. The results of our study showed that middle-aged and older adult people who used the Internet were less likely to report loneliness than those who did not use the Internet, which is consistent with the results of Heo, Xu, Khalaila and others (43–45). In addition, the COVID-19 pandemic has reinforced the role of the Internet in reducing loneliness during social distancing (46). The reasons are as follows. On the one hand, the Internet has the advantages of real-time convenience and convenient interaction, which can break the time and space constraints, so that the older adult can keep close contact with relatives, friends and children (47). This way of communication plays an important role in delaying anxiety, improving the sense of belonging and reducing loneliness (48). On the other hand, the Internet provides an online social environment for the older adult (49), which can establish a harmonious social network relationship by meeting new friends, promote and enhance the social adaptability of the older adult (50). In addition, spending time on the Internet can enrich the leisure activities of the older adult, meet their social needs with the outside world, obtain external information, expand social interaction space, help to improve their life satisfaction (51, 52), and thus regulate individual psychological state (7).

However, residence played a moderating role in the relationship between Internet use and loneliness, and only middle-aged and older adult people in urban areas felt significantly less lonely when using the Internet. On the one hand, the main reason is that the Internet penetration rate, network infrastructure construction and other hardware conditions in rural areas lag behind those in urban areas. The development of the Internet in China’s urban areas is faster than that in rural areas. The Internet penetration rate in urban and rural areas is 81.3 and 57.6%, respectively. The scale of Internet users in rural areas still lags behind that in cities, and the middle-aged and older adult people in cities have more convenient access to the Internet. The cost and speed of Internet access may affect the willingness of the older adult people in rural areas to use the Internet (9, 25). On the other hand, due to the long-term impact of China’s urban–rural dual structure, rural and urban residents are different in many aspects, including education, income, available resources, interpersonal relationships and living communities (32). Middle-aged and older adult people living in urban communities have a relatively closed living environment, and independent high-rise buildings restrict their social activities. However, community management organizations provide a higher level of public services, and they are more likely to use the Internet to participate in community activities to alleviate loneliness. Compared with cities, villages where rural residents live are more in line with the traditional way of “living in groups” and have close interpersonal communication. And the rural older adult do not have a fixed retirement age, they are still engaged in agricultural work after the age of 60, enriching their daily activities, the Internet has little impact on their lives, and has not played a role in alleviating loneliness (53).

Thirdly, for the middle-aged and older adult people who use the Internet, there are significant differences in the impact of different Internet use time and content on loneliness. In terms of the duration of Internet use, compared with the older adult who use the Internet for less than 1 h a day, the older adult who use the Internet for 1–3 h and 3–5 h a day have less risk of loneliness, while the duration of Internet use for more than 5 h is not statistically significant. Although there is a negative correlation between Internet use and presence loneliness, overuse may offset its positive effects, reduce communication in real life, increase the sense of isolation between people, and have a negative impact on their mental health (25, 54). In terms of the content of Internet use, different contents have different effects on the loneliness of middle-aged and older adult people. Middle-aged and older adult people who use the Internet to contact with family and friends, read the news and manage money were less likely to report loneliness. This conclusion is consistent with the findings of Jin and Choi, whose research results show that the leisure provided by the Internet enriches people’s lives (55), and that social software such as Weixin, Weibo and QQ can improve the level of social interaction, thereby alleviating loneliness (29). In addition, our study also found that middle-aged and older adult people who use the Internet for online payment were more likely to report loneliness. Cham’s research showed that most seniors were skeptical about using online payments (56). First, they lack sufficient trust in network security and worry about the leakage of personal information or the loss of personal property. Second, the complexity of Internet technology may make them anxious about technology use. This also confirms the research of Erceg et al., who believe that certain online activities can lead to compulsive use of the Internet, resulting in higher levels of anxiety and loneliness, affecting mental health (57).

The advantage of this study is that it further confirms the negative relationship between Internet use and loneliness in middle-aged and older adult people, explores the role of residence in it, and provides new evidence for the relationship between the length and content of Internet use and loneliness. The study also has some limitations. Firstly, this study used only one self-reported item to measure loneliness. Because the survey time should not be too long during the epidemic period, and the small sample size of the survey limited the length of the questionnaire, we did not design too many questions. In the design of the questionnaire, we refer to a large-scale survey in China, the health status survey data of China’s older adult population (CLHLS), to measure loneliness by “do you feel lonely.” Which can only explore the relationship between Internet use and the presence of loneliness, but can not measure the degree of loneliness. Moreover, the inclusion of other control variables in the study was limited. In the future, the Likert scale should be used to measure the intensity, duration, and frequency of loneliness in subsequent studies, and include as many control variables as possible, taking into account family characteristics, social support, health status and other factors of middle-aged and older adult people. Secondly, the explanation of the difference in the moderating effect of residence in this study is only an empirical and theoretical inference, and the deeper mechanism behind the phenomenon needs additional special discussion. Third, due to the limitation of data, there may still be some endogenous problems that are difficult to eliminate in the study process. The cross-sectional studies cannot determine causality, so the results presented in this study cannot answer whether Internet use contributes to the key variables of loneliness in middle-aged and older adult people. We will further analyze the relationship between Internet use and loneliness in future studies.

This study revealed that middle-aged and older people who used the Internet were less likely to report loneliness. In addition, different types of Internet use content have different effects on loneliness, and contacting relatives and friends, watching news and managing money were related to lower loneliness, while online payment was related to higher loneliness. At the same time, the longer they used the Internet, the less likely they were to report loneliness, but overuse may have a negative impact. In general, the impact of Internet use on middle-aged and older adult people has two sides. On one hand, Internet use gives possibility to reduce the presence of loneliness and mental-health improvement. On other, there is also a risk of Internet addiction when using the Internet excessively for a long time. Connection between loneliness and Internet addiction could be a direction of the future studies. Therefore, we should continue to pay attention to the relationship between Internet use and loneliness of middle-aged and older adult people. Manufacturers can develop smart products for aging, which are convenient for their daily operation and use, and reduce the barriers for them to use the Internet. Family members should encourage and guide them to actively use the Internet to strengthen social interaction, and pay attention to the time of surfing the Internet. Society should establish a good network environment to protect their safe use of the network and avoid telecommunications fraud, personal information leakage and other incidents.

## Data availability statement

The datasets generated and analyzed during the current study are not publicly available as data analysis has not been completed but is available from the corresponding author on reasonable request.

## Ethics statement

Written informed consent was obtained from all participants in this study. This study was approved by the Ethics Committee of Weifang Medical University.

## Author contributions

QL: Data curation, Investigation, Writing – original draft, Conceptualization, Methodology, Writing – review & editing. ChuY: Conceptualization, Data curation, Writing – review & editing, Investigation, Methodology, Writing – original draft. ZZ: Conceptualization, Data curation, Investigation, Methodology, Writing – original draft, Writing – review & editing. CheY: Data curation, Investigation, Methodology, Writing – original draft, Writing – review & editing. ZC: Conceptualization, Data curation, Investigation, Methodology, Writing – original draft, Writing – review & editing. DH: Conceptualization, Funding acquisition, Methodology, Supervision, Writing – original draft, Writing – review & editing. WY: Conceptualization, Data curation, Funding acquisition, Writing – original draft, Writing – review & editing.
